# Toxic oligomers of the amyloidogenic HypF-N protein form pores in mitochondrial membranes

**DOI:** 10.1038/s41598-020-74841-z

**Published:** 2020-10-20

**Authors:** Maria Ylenia Farrugia, Mario Caruana, Stephanie Ghio, Angelique Camilleri, Claude Farrugia, Ruben J. Cauchi, Sara Cappelli, Fabrizio Chiti, Neville Vassallo

**Affiliations:** 1 Department of Physiology and Biochemistry, Faculty of Medicine and Surgery, University of Malta, Msida, Malta; 2 Centre for Molecular Medicine and Biobanking, University of Malta, Msida, Malta; 3 Department of Chemistry, University of Malta, Msida, Malta; 4 Section of Biochemistry, Department of Experimental and Clinical Biomedical Sciences, University of Florence, 50134 Florence, Italy

**Keywords:** Ion channels, Lipids, Neurochemistry, Protein folding

## Abstract

Studies on the amyloidogenic N-terminal domain of the *E. coli* HypF protein (HypF-N) have contributed significantly to a detailed understanding of the pathogenic mechanisms in neurodegenerative diseases characterised by the formation of misfolded oligomers, by proteins such as amyloid-β, α-synuclein and tau. Given that both cell membranes and mitochondria are increasingly recognised as key targets of oligomer toxicity, we investigated the damaging effects of aggregates of HypF-N on mitochondrial membranes. Essentially, we found that HypF-N oligomers characterised by high surface hydrophobicity (type A) were able to trigger a robust permeabilisation of mito-mimetic liposomes possessing cardiolipin-rich membranes and dysfunction of isolated mitochondria, as demonstrated by a combination of mitochondrial shrinking, lowering of mitochondrial membrane potential and cytochrome *c* release. Furthermore, using single-channel electrophysiology recordings we obtained evidence that the type A aggregates induced currents reflecting formation of ion-conducting pores in mito-mimetic planar phospholipid bilayers, with multi-level conductances ranging in the hundreds of pS at negative membrane voltages. Conversely, HypF-N oligomers with low surface hydrophobicity (type B) could not permeabilise or porate mitochondrial membranes. These results suggest an inherent toxicity of membrane-active aggregates of amyloid-forming proteins to mitochondria, and that targeting of oligomer-mitochondrial membrane interactions might therefore afford protection against such damage.

## Introduction

Neurodegenerative diseases of the amyloid type, such as Alzheimer’s disease (AD) and Parkinson’s disease (PD), are thought to be driven by the self-assembly of polypeptide chains into a variety of misfolded conformers along an aggregation continuum^1,2^. Multiple lines of evidence now point to small soluble oligomers formed early in the aggregation process as being the main culprit to cause neurotoxicity. Over the past two decades or so, much research effort has been expended to uncover the structure, mechanism of formation, and biological activity of misfolded protein oligomers^3,4^.

One of the key questions that remain unanswered concerns the structural elements of oligomers that are responsible for their toxicity^1,5^. In this regards, the 91-residue *Escherichia coli* amyloid-forming HypF-N protein, which corresponds to the N-terminal domain of the prokaryotic hydrogenase maturation factor HypF and which has no link to human disease, has proved to be a highly valuable model system. Monomeric HypF-N under suitable in vitro conditions can be induced to form spherical oligomers, protofibrils and amyloid-like fibrils, with the oligomeric species being highly cytotoxic if added to the extracellular medium of cultured cells—unlike mature fibrils or monomers, which remain relatively benign^6–11^. A most experimentally-useful feature of the HypF-N aggregating model is that oligomer formation can be easily directed into two structurally similar forms, referred to as type A and type B, by simply altering the incubation conditions. The two oligomer types generated under conditions A and B are both highly stable, appear roughly spherical under atomic force microscopy with a diameter of 2–6 nm, and have a similar β-sheet core structure displaying weak Thioflavin-T (Th-T) binding^11–13^. However, the two species differ in one critical aspect: whereas type A oligomers induce dysfunction of cells, type B oligomers are harmless. Type A oligomers manifested a strong ability to penetrate cell membranes and initiate a series of downstream events associated with cytotoxicity, including an influx of Ca^2+^ ions from the cell medium, increase in intracellular reactive oxygen species generation and lipid peroxidation^11,13^. Strikingly, type A oligomers also induced loss of cholinergic neurons when microinjected into rat brains with an associated impairment of spatial memory and inhibition of long-term potentiation (LTP) in rat hippocampal slices, thereby mimicking toxicity of the 42-residue amyloid-β peptide (Aβ_42_) associated with AD^13,14^.

One common theme shared by neurodegenerative proteinopathies when they are studied in vitro is the interaction of toxic protein aggregates with lipid species. Aggregated protein assemblies, including prefibrillar oligomers, protofibrils and amyloid fibrils, may compromise bilayer membrane integrity by various mechanisms, such as membrane thinning, membrane poration or lipid extraction, resulting in functional impairment of the cell^15–18^. A study in rats has provided some evidence that misfolded oligomer-membrane interactions may also be relevant in vivo—expression of highly membrano-toxic variants of PD-associated α-synuclein in rats correlated with the highest dopaminergic neuronal loss in the substantia nigra^19^.

Crucially, the composition and physicochemical properties of a membrane strongly influence peptide/protein insertion and aggregation. The two principle mechanisms involve electrostatic interactions between the polypeptide chain and lipid head groups, and hydrophobically-driven insertion. For example, the Aβ_42_ peptide has a net negative charge at physiological pH, which would suggest repulsion at anionic membrane surfaces. Here, the hydrophobic Aβ(25–35) domain is accredited for being essential in the membrane anchoring of the full-length protein with the lipid membrane, leading to aggregation and packing of the acyl tails in the hydrophobic membrane core^20,21^. For cationic proteins such as tau and the N-terminal domain of α-synuclein, negatively-charged membrane surfaces drive electrostatic attraction between basic side chains in the protein and lipid head groups or gangliosides in the external membrane leaflet, facilitating the formation of toxic aggregates in situ at the membrane^22–27^. Likewise, prefibrillar assemblies of HypF-N were shown to preferentially permeabilise bilayers enriched in anionic phospholipids or glycolipids^28–30^. A unique lipid composition is found in the double-membrane envelope of mitochondrial organelles, featuring outer (OMM) and inner (IMM) mitochondrial membranes^31^. The IMM is particularly abundant in cardiolipin (CL), a signature lipid of energy-generating membranes synthesised from phosphatidylglycerol (PG) through cardiolipin synthase (CLS1) and the membrane-acting enzyme tafazzin (TAZ), which remodels the acyl chains^32,33^. CL plays a pivotal role in bioenergetics, cristae morphology and the organisation of components of the electron transport chain into respiratory supercomplexes^34,35^. CL is also enriched at mitochondrial contact sites, where the OMM and IMM are in close apposition with each other, allowing CL to diffuse from the inner to the outer membrane^36,37^. CL has a unique di-phosphatidylglycerol structure, where two phosphatidic acids form phosphodiester bonds with a glycerol residue that bridges them, resulting in a dimeric arrangement and four acyl chains. The overall structure of CL is thus conical, with a small head group carrying two negative charges (one on each phosphate group) and a large hydrophobic tail^35^. To some extent, CL molecules impart mechanical membrane instability as they lead to a decrease in the packing capability of the lipids (non-lamellar formation) and an increase in membrane fluidity^38^.

In the present investigation, we made use of the HypF-N model system to answer the question as to whether pathogenic protein oligomers might be uniquely predisposed to perturb mitochondrial membranes with high CL content (15–20% CL). To help unravel the mechanistic processes and better understand the complex cellular system, we adopted a reductionist approach consisting of synthetic model membranes and isolated mitochondria^39^.

## Results

### Permeabilisation of mito-mimetic lipid vesicles by HypF-N aggregate species

In order to explore the potential of aggregates from the *E.coli* amyloidogenic HypF-N protein to perturb mitochondrial membranes, we first employed an in vitro reconstituted system consisting of biomimetic large unilamellar lipid vesicles (LUVs) loaded with the fluorophore Oregon Green 488 BAPTA-1 (OG). LUVs have been employed in many biophysical studies as the simplest model membrane to reveal fundamental information about protein-lipid interactions^40,41^. By following the OG fluorescence over time, we could observe the evolution of LUV leakage induced by HypF-N at different states of aggregation. In particular, we compared the permeabilisation efficiency of the two known stable oligomeric forms of HypF-N, the toxic type A and non-toxic type B oligomers. HypF-N species were applied to OG-loaded LUVs having either a CL-enriched mitomimetic membrane reflecting the composition of the IMM and mitochondrial contact sites (IM-type: 45PC/25PE/10PI/5PS/15CL), or a phospholipid blend reflecting the composition of neuronal and synaptic vesicle membranes (C-type: 50DOPE/30DOPS/20DOPC).

**Figure 1:**
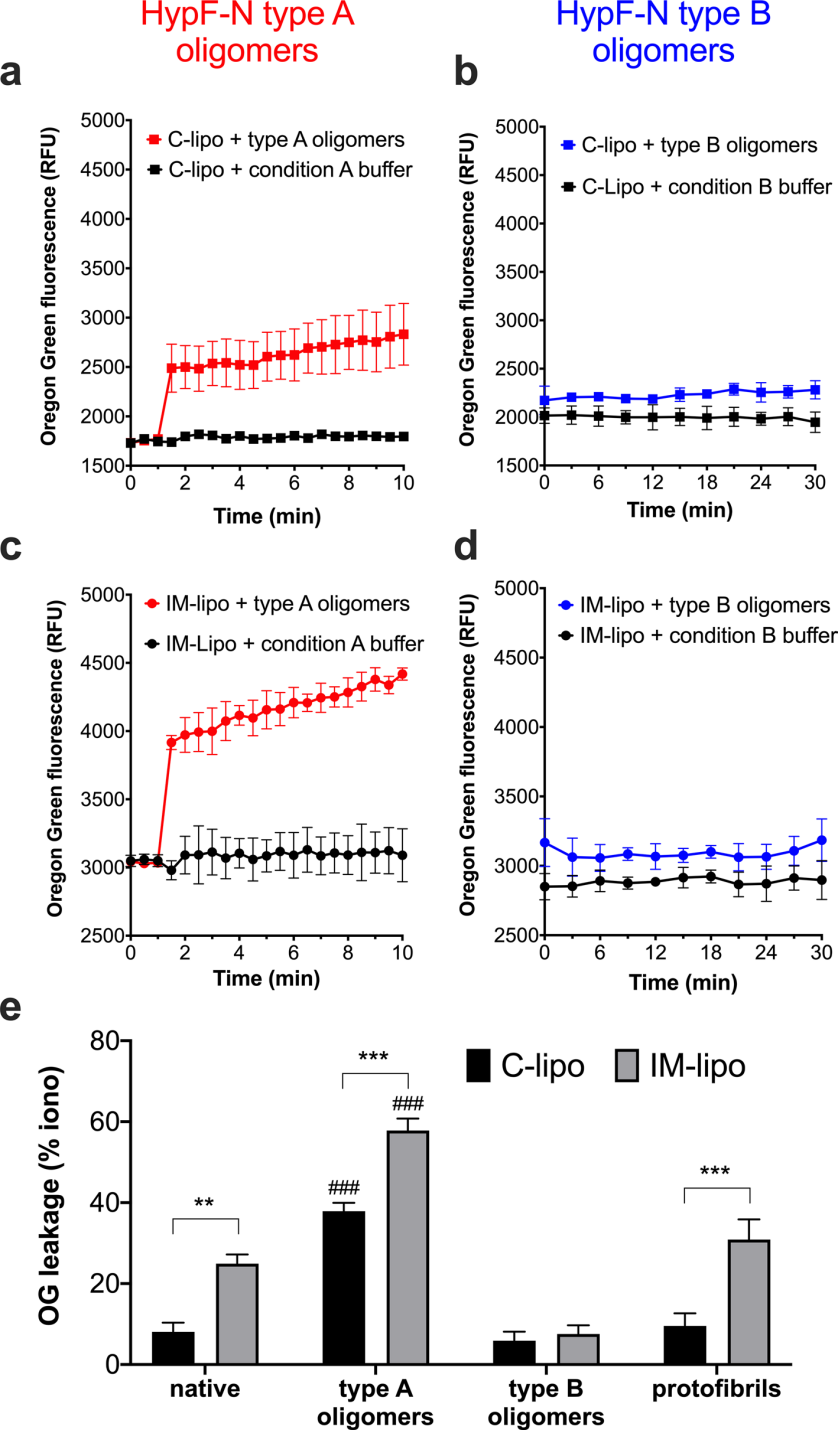
Permeabilisation kinetics and leakage of LUVs having mito-mimetic (IM-type) and neuronal-like (C-type) membranes induced by HypF-N protein in different aggregation states. (**a–d**) Time dependence of fluorescence of OG entrapped in C-type (C-Lipo) or IM-type (IM-Lipo) lipid vesicles following addition of 2 μM HypF-N oligomers (type A or type B) at a peptide-to-lipid molar ratio of 1:25. Control liposomes without peptide were incubated in liposome buffer containing equivalent concentrations of condition A or condition B aggregation buffer. Error bars represent the s.e.m. of 3 replicate experiments. (**e**) Comparison of OG dye leakage from C-lipo and IM-lipo by different HypF-N species. Leakage by monomeric (native), oligomeric (type A or type B oligo) and protofibrillar HypF-N are shown as a percentage of that induced by the Ca^2+^ ionophore ionomycin (iono). Data are presented as means ± s.e.m. (*n* = 3–7); ** *p* < 0.01, *** *p* < 0.001, between marked pairs; ### *p* < 0.001, between type A and type B oligomers (two-way ANOVA with Bonferroni’s correction).

Addition of type A HypF-N oligomeric aggregates to both IM-type and C-type LUVs resulted in a sharp elevation of OG fluorescence over 30 s, followed by a more gradual and constant rise in fluorescence over the 10-min duration of the permeabilisation experiment (Fig. 1a,c). The distinct two-step kinetic process for membrane perturbation by HypF-N type A oligomers recalls similar mechanisms previously reported for the Aβ_42_ peptide, suggesting early permeabilisation by pores followed by membrane disruption by a detergent-like mechanism^42^. Of note, permeabilisation of IM-liposomes by type A oligomers was around 28% higher than that of C-liposomes after 10 min (1328 RFU vs.1032 RFU, respectively), indicating a greater susceptibility of the mito-mimetic LUVs to oligomer-induced membrane damage. In contrast, the aggregation buffer (condition A) alone did not exhibit any increase in fluorescence signal, thereby serving as a control for the effect of buffer. Furthermore, a negative control of type A HypF-N oligomers incubated with IM-liposomes without encapsulated fluorophore yielded no appreciable fluorescence (data not shown).

Remarkably, the non-toxic type B HypF-N oligomers did not instigate any appreciable loss of integrity in either the C-type or the IM-type lipid vesicles, even after 30 min of incubation (Fig. 1b,d). The liposome permeabilisation assay was also carried out with native (monomeric) and protofibrillar HypF-N species, again using C-type liposomes as well as IM-type liposomes. A direct comparison was made between the maximal OG leakage induced by the different HypF-N species on C-liposomes and IM-liposomes, calculated as a percentage of that triggered by addition of the (non-fluorescent) Ca^2+^ ionophore ionomycin to the vesicles (theoretical 100%) (Fig. 1e). For liposomes with C-type membranes, the only significant permeabilisation was induced by the type A oligomers [37.8 ± 2.0%, *p* < 0.0001 with respect to native monomeric (8.0 ± 2.3%), type B oligomeric (5.9 ± 2.3%) and protofibrillar (9.5 ± 3.1%) HypF-N species]. Type A oligomers were also the most membrane-damaging HypF-N species on liposomes with IM-type membranes [57.8 ± 3.0%, *p* < 0.0001 with respect to native monomeric (25.0 ± 2.3%), type B oligomeric (7.6 ± 2.2%) and protofibrillar (30.9 ± 5.0%) HypF-N species]. Notably, native, type A oligomeric and protofibrillar HypF-N were damaging only to IM-liposomes (Fig. 1e), signifying a preference for HypF-N for compromising the integrity of CL-rich membranes.

**Figure 2:**
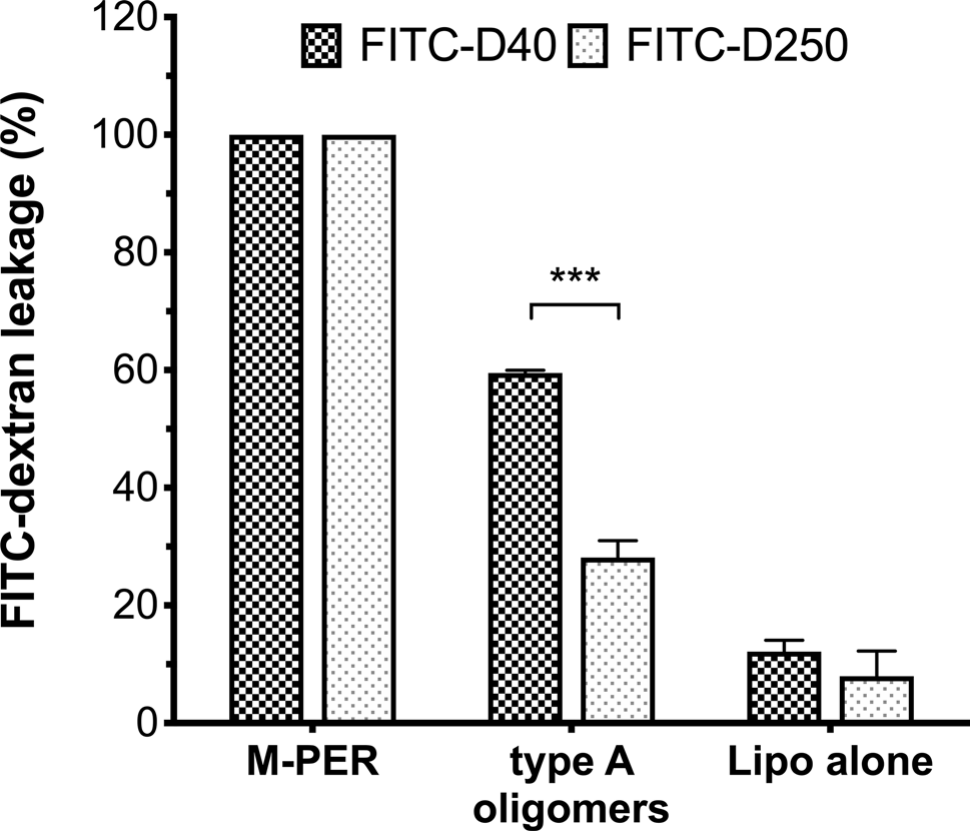
Leakage of FITC-dextran polymer (40 kDa and 250 kDA) from liposomes by HypF-N type A oligomers. Maximum leakage of FITC-40 and FITC-250 dextran molecules from 50 μM IM-type liposomes was determined after the addition of 2 μM type A HypF-N oligomeric aggregates, and is shown as a percentage of that induced by the detergent M-PER. Also shown is the background leakage from the liposomes incubated with an equivalent concentration of condition A aggregation buffer alone, to control for the effect of buffer. Data are presented as means ± s.e.m. (*n* = 5); *** *p* < 0.0001 (two-way ANOVA with Bonferroni’s correction).

Since the two-step kinetic leakage profile for type A oligomers was consistent with the initial formation of membrane pores, we studied the ability of HypF-N type A oligomers to induce leakage of different-sized fluorescence probes. We therefore used fluorescein isothiocyanate (FITC) conjugated to dextrans of 40 kDa (FITC-D40) and 250 kDa (FITC-D250) and determined FITC leakage from LUVs composed of IM-type membrane, after incubation with type A oligomers. Indeed, HypF-N type A oligomers lead to a strong efflux of FITC-dextran, with leakage of FITC-D40 molecules occurring to a much greater extent (~ twofold higher) than leakage of FITC-D250 molecules, calculated as a percentage of maximal leakage induced by the M-PER detergent (59.5 ± 0.5% for FITC-D40 *vs.* 29.2 ± 3.4% for FITC-D250, *p* < 0.0001). As expected, the IM-type LUVs incubated with condition A buffer alone (i.e. without HypF-N protein) remained stable throughout the duration of the experiment, with only a slight leakage of FITC-dextran (Fig. 2). The size-dependent leakage implies that supramolecular pore formation likely plays a role in the permeabilisation of mito-mimetic lipid membranes by toxic type A oligomers.

### Permeabilisation of isolated mitochondria by HypF-N aggregate species

Having demonstrated the propensity of type A HypF-N oligomers to enhance mitochondrial-like membrane permeability in LUVs, we next wanted to extrapolate our findings to biomembranes, i.e. the membranes of mitochondrial organelles. Thus, HypF-N aggregate species were applied directly to freshly respiring mitochondria, isolated from human neuroblastoma SH-SY5Y cells. Three assays were performed on the mitochondria, namely: measurement of changes in mitochondrial volume, quantification of release of cytochrome *c* (cyto *c*), and determination of the inner mitochondrial membrane potential (ΔΨ_m_). Collectively, these assays provide a useful ‘toolbox’ for assessing perturbation of mitochondrial membrane integrity by HypF-N.

**Figure 3:**
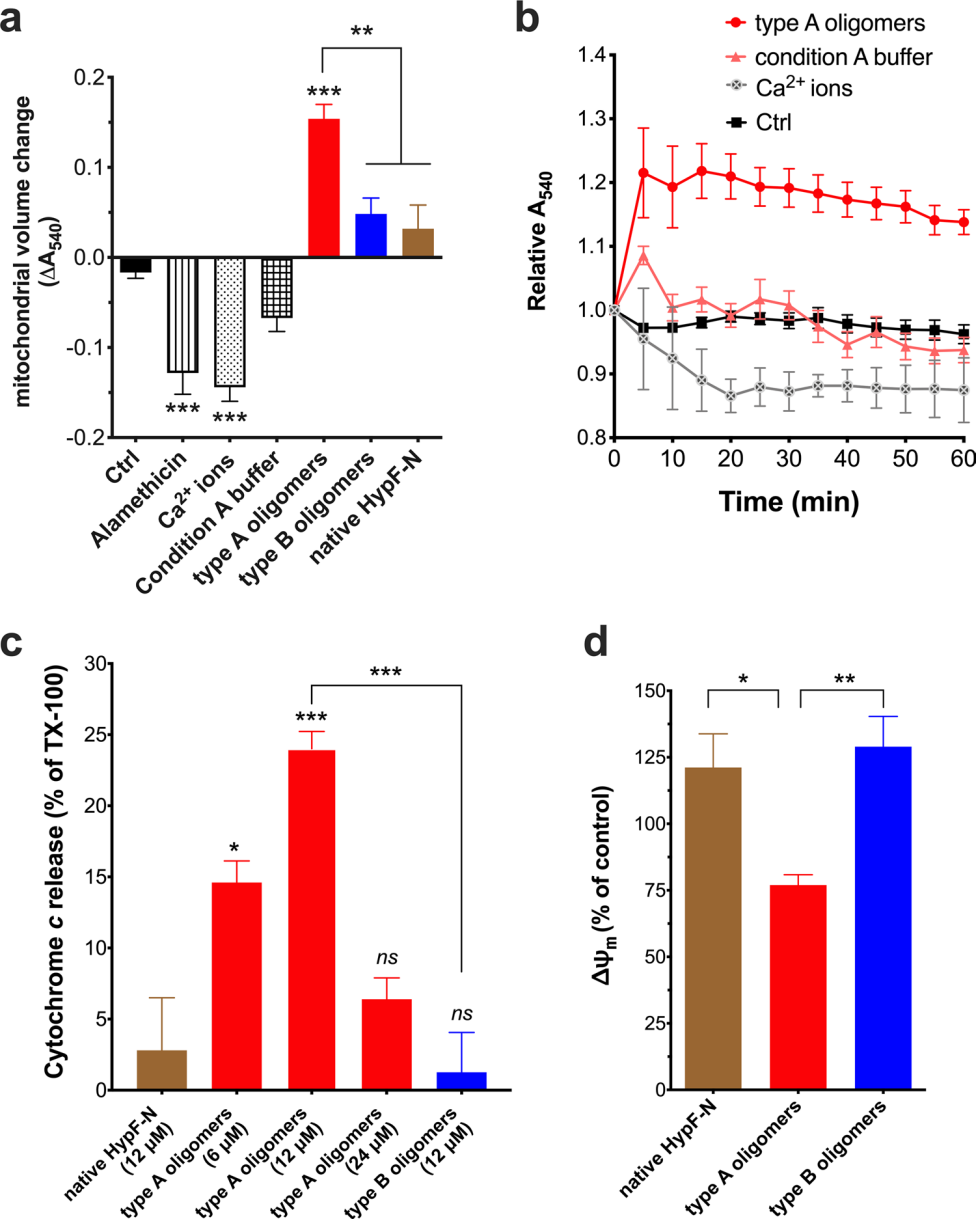
Effects of HypF-N species on organelle volume, cytochrome *c* release and Δψ_m_ of isolated mitochondria. (**a**) Changes in the absorbance at 540 nm (A_540 nm_) in a suspension of freshly isolated mitochondria in MB after 60 min incubation, alone (Ctrl) or with the swelling-inducing agents 50 μM alamethicin and 250 μM CaCl_2_ (Ca^2+^), and with 10 μM HypF-N in monomeric (native) and oligomeric (type A, type B) states. Condition A buffer alone (used to generate the type A oligomers) was also included as a control. Significant swelling of mitochondria is triggered by alamethicin and Ca^2+^, as expected, whilst type A oligomers cause organelle shrinkage. (**b**) Representative kinetic traces (*n* = 3–5) showing the absorbance measurements (A_540 nm_), relative to the value at time 0 min, every 5 min for mitochondria incubated alone (Ctrl) or with 250 μM CaCl_2_ (Ca^2+^ ions), condition A buffer, and 10 µM HypF-N type A oligomers. The shrinkage effect of the type A aggregates is immediate and maintained over 60 min. (**c**) Effect of HypF-N species on the cyto *c* release (CCR) from isolated mitochondria incubated in MB for 60 min, given as a percentage of 1% (*v/v*) Triton X-100 (TX-100). Amount of cyto *c* released into buffer was determined using Quantikine immunoassay (~ 35 ng/ml with TX-100). A significant CCR was evoked by type A, but not type B or native HypF-N, at the same concentrations (12 μM). (**d**) Measurement of Δψ_m_ by JC-1 dye, from isolated mitochondria following 30-min exposure to equimolar concentrations (2 μM) of HypF-N (native, type A and type B oligomers). Δψ_m_ is given as a percentage of untreated mitochondria control (100%), after baseline correction obtained by dissipating Δψ_m_ using FCCP. A reduction in the Δψ_m_ is observed in the case of type A oligomers. In all panels data are presented as means ± s.e.m. (*n* = 3–6, with duplicate or triplicate measurements); * *p* < 0.05, ** *p* < 0.01, *** *p* < 0.001, with respect to Ctrl or native HypF-N, or between marked pairs (one-way ANOVA with Bonferroni’s correction).

A convenient and frequently used assay to measure changes in mitochondrial volume is to monitor the absorbance at 540 nm (A_540_) of a purified mitochondrial preparation, wherein a decrease (or increase) in A_540_ reflects an increase (or decrease) in light transmission, which in turn corresponds to an increase (or decrease) in mitochondrial volume^43^ (Fig. 3a). Mitochondria in buffer alone remained stable for the 60-min duration of the experiment (− 0.017 ± 0.001 U). To induce mitochondrial swelling as positive controls, the pore-forming peptide alamethicin^44^ and Ca^2+^-induced opening of the permeability transition (PT) pore^45^ were used. In fact, both mediated a significant degree of mitochondrial swelling after 60 min as measured by a decrease in the relative A_540_ (alamethicin: − 0.128 ± 0.024 U, *p* < 0.0001; Ca^2+^ ions: − 0.144 ± 0.016 U, *p* < 0.0001). Isolated mitochondria exposed to type A HypF-N oligomers underwent a significant increase in A_540_ (+ 0.154 ± 0.016 U, *p* < 0.0001) which indicates that shrinking of the mitochondrial organelle had occurred^46^. Condition A buffer alone, on the other hand, induced only a temporary increase in A_540_ which was followed by a progressive stabilisation of mitochondrial volume over the next 40 min to control absorbance values (− 0.067 ± 0.015 U, *p* = 0.1484). Further, neither native non-aggregated, nor type B aggregated, HypF-N protein caused any significant change in mitochondrial volume (native: + 0.032 ± 0.026 U, *p* = 0.1484; type B oligomers: + 0.048 ± 0.018 U, *p* = 0.1094), thus nicely reflecting the negative results of the LUV permeabilisation assays. Looking at the kinetic traces, it can be observed that an increase in the relative A_540_ (i.e. mitochondrial shrinking) occurred within 5 min of incubation of the mitochondria with the HypF-N type A oligomers (1.215 ± 0.07 U at *t* = 5 min), which was maintained over the course of the experiment (1.138 ± 0.02 U at *t* = 60 min) (Fig. 3b). In conclusion, HypF-N type A oligomers caused an immediate shrinking of mitochondria that was maintained for up to 1 h.

Shrinkage of the mitochondrial matrix has been associated with triggering of cyto *c* efflux from mitochondria and an inhibition of mitochondrial respiration^47^. Therefore, we proceeded to determine whether this is also the case when isolated mitochondria are incubated with HypF-N type A oligomers, and compare the effect of type A with type B oligomers and native species (Fig. 3c). We observed pronounced cyto *c* release (CCR) with type A oligomers at 6 μM (14.6 ± 1.5%, *p* = 0.0183) and 12 μM (24.0 ± 1.2%, *p* = 0.0003) with respect to native HypF-N (2.8 ± 3.7%). At a very high concentration of 24 μM, however, CCR by type A oligomers was not significantly different from native HypF-N (6.4 ± 1.5% vs. 2.8 ± 3.7%, respectively). This is likely due to agglutination or further acceleration of oligomer aggregation during incubation into a fibrillar, less toxic species. Importantly, CCR mediated by 12 μM type B oligomers (1.3 ± 2.8%) was not significantly different from that induced by equimolar concentrations of native HypF-N, indicating that triggering of CCR is specific to cytotoxic type A oligomers (*p* = 0.0002, type A vs. type B).

Another indicator of intact mitochondrial membranes, and an important physiological parameter of mitochondrial function, is the voltage gradient across the inner membrane (ΔΨ_m_). The ΔΨ_m_ was tracked over 30 min during application of native, type A and type B HypF-N oligomers to isolated mitochondria, by observing the uptake of the cationic carbocyanine dye JC-1 into the mitochondrial matrix (Fig. 3d). Again, only type A oligomers caused a significant decrease in ΔΨ_m_ with respect to monomeric HypF-N (type A: 77 ± 4%, *p* = 0.0276; type B: 129 ± 11%; native: 121 ± 12%). Taken together, the data clearly show that type A HypF-N oligomers are potent mito-toxins, in line with their ability to permeabilise mito-mimetic, CL-enriched membranes of lipid vesicles.

### Electrophysiological characterisation of channel activity induced by type A HypF-N oligomers in planar lipid bilayer membranes

**Figure 4:**
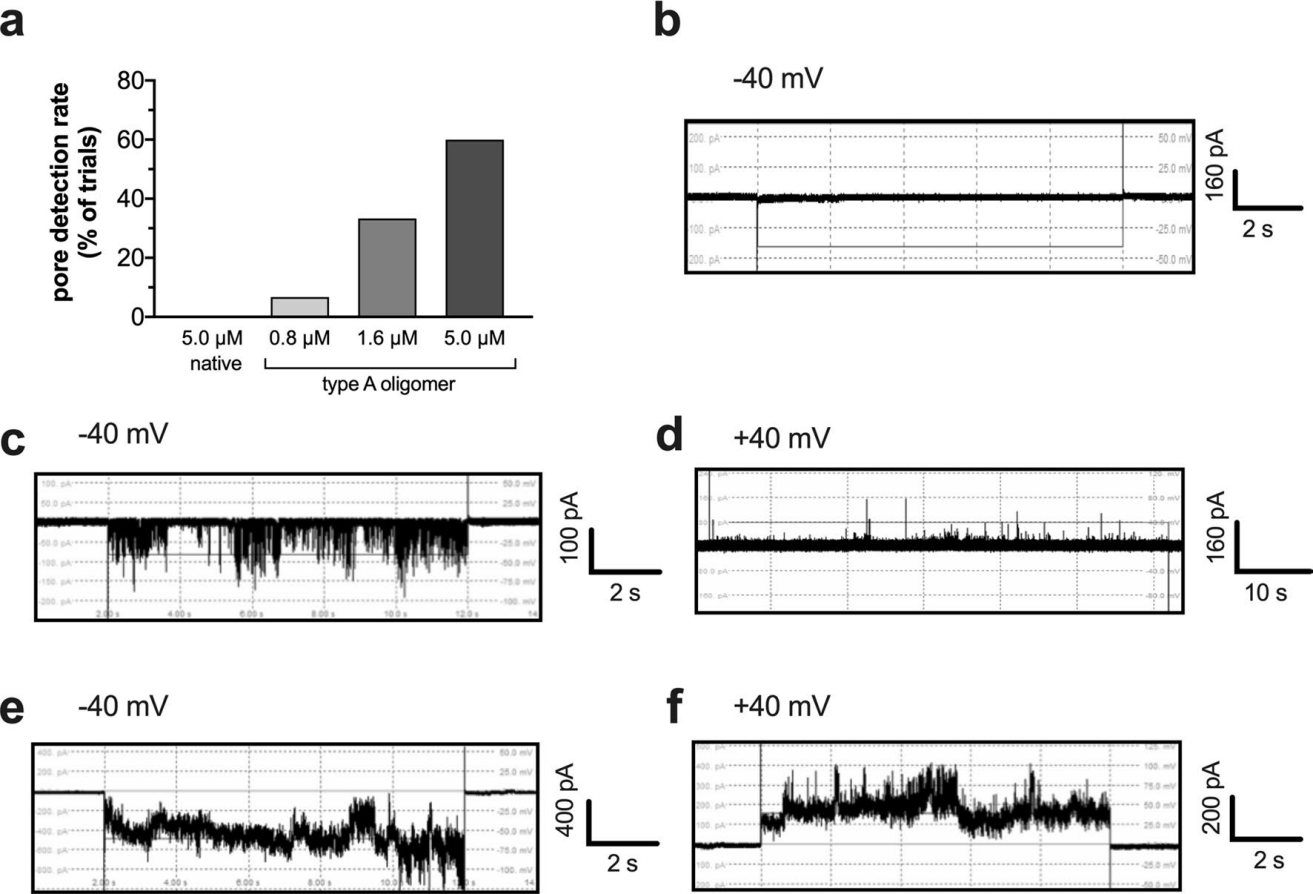
Electrophysiological characterisation of type A HypF-N pores in IM-type planar lipid bilayer. (**a**) Success rate of pore formation for each HypF-N species type, as a percentage of the total number of electrophysiology trials (*n* = 10 for 5 μM native HypF-N, and* n* = 15, 12 and 10 for 0.8 μM, 1.6 μM and 5 μM HypF-N type A oligomers, respectively). (**b**) Control trace devoid of channel activity, when native HypF-N or condition A aggregation buffer were added to the *cis-*chamber. (**c–f**) Representative current traces after addition of 5 µM oligomeric type A HypF-N. Typical current recordings are shown for type-I (**c,d**) and type-II (**e,f**) pores, at square wave-voltage pulses of − 40 mV and + 40 mV. The induced currents are indicative of the formation of HypF-N nanopores in the BLM. Electrical recordings were all carried out on IM-type planar BLM (45 PC/25 PE/10 PI/5 PS/15 CL) under symmetrical buffer conditions (250/250 mM KCl, 10 mM MOPS/Tris, pH 7.2), using PatchMaster standard protocols.

We next explored whether the capacity of type A HypF-N oligomers to compromise the integrity of mitochondrial membranes, was associated with their ability to form ion-conducting channels in CL-rich lipid bilayers. To this end, we made use of the planar bilayer lipid membrane (BLM) technique, which is an extremely sensitive method for elucidating whether reconstitution of purified proteins and peptides in artificial lipid bilayers leads to channel-like current activity across the membrane^48^. In order to align the electrophysiology experiments with the liposome permeabilisation assays, the phospholipid components of the BLM were the same as those of the LUV membranes, i.e. IM-type. After ensuring a stable bilayer for at least 15 min, 5 μM HypF-N protein (native, type A, and type B) were added to the *cis-*side of the bilayer and changes in the permeability of the planar BLM monitored for up to 2 h, at a clamped voltage of ± 50 mV. Channel-like activity (conductance > 70 pS) was detected in 60% of electrophysiology trials (pore detection rate: *n* = 6 out of 10 trials) when aliquots containing pre-formed type A aggregates (5 μM) were applied (Fig. 4a). Application of lower concentrations of type A oligomers led to a decrease in the pore detection rate, in a concentration-dependent manner: 1.6 μM, 33% (*n* = 4 out of 12 trials); 0.8 μM, 6.7% (*n* = 1 out of 15 trials). This concentration-dependency further supports the notion that the BLM activity detected upon addition of aliquots of the type A oligomer preparation was indeed a direct consequence of protein-based membrane incorporation events. In contrast, neither type B aggregates, nor native monomeric HypF-N, nor condition A aggregation buffer lead to any increased current activity at all tested incubation times (*n* = 0 out of 10 trials each) (Fig. 4a,b). As a positive control for pore formation in lipid bilayer, the *Staphylococcus aureus* α-haemolysin toxin was tested, which invariably formed a distinct oligomeric pore (data not shown).

Following addition of 5 μM type A oligomers to the *cis-*chamber, the median time elapsed for pore activity to be first detected in the bilayer was 41 min (range: 25–150 min). From the current traces, it was observed that, based upon the general characteristics of the single-channel recordings, two distinct types of pore activity by the type A oligomers could be distinguished. Thus, ‘type-I’ pore activity was represented by a continuous, ‘flickering’ behaviour (Fig. 4c,d), while ‘type-II’ pore activity was seen as reflecting more ‘step-like’ jumps in current (Fig. 4e,f). Type-II behaviour was seen in all traces, whenever HypF-N current activity from type A oligomers was observed. Type-I electrical signals occurred in only a third of experiments (*n* = 2 out of 6 positive trials) and were never seen alone, always occurring in the initial traces prior to the subsequent observation of type-II signals. Intriguingly, a similar pattern of noisy transitions together with reasonably well-defined conductance behaviour was also seen in electrical recordings of β-barrel pore-forming Aβ_42_ oligomers incorporated into planar lipid bilayers^49^.

**Figure 5:**
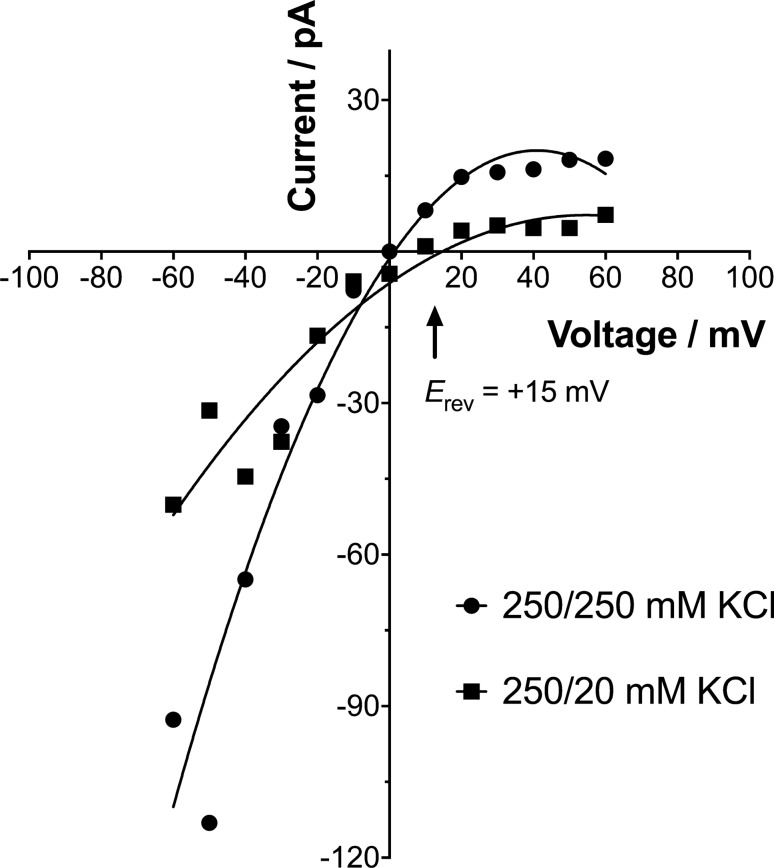
Current–voltage (*I–V*) relationships for a single HypF-N pore. Current–voltage ramp was performed under symmetric (250/250 mM KCl) and asymmetric (250/20 mM KCl) buffer conditions (10 mM MOPS/Tris, pH 7.2, 22 °C) whilst a type A HypF-N pore was inserted in the IM-type bilayer membrane. The intercept on the *V* axis equivalent to the reversal potential (*E*_*rev*_) is given at + 15 mV, implying a selectivity for cations. The *I–V* data set is fitted to a second order polynomial (quadratic) equation using a least squares fit (*n* = 2 independent experiments).

We then proceeded to carry out an electrophysiological characterisation of the pore events evoked by type A HypF-N oligomers (at 5 μM) in the mitochondrial-mimetic BLM, including a detailed analysis of the conductance states and dwell times, estimation of pore diameter, current–voltage (*I-V*) relationship curve, determination of the reversal potential (*E*_*rev*_) and ion selectivity. Thus, *I–V* relationship curves were constructed by plotting the value of the current recorded for each clamped membrane potential (from − 60 mV to + 60 mV) under symmetric (250/250 mM KCl, 10 mM MOPS/Tris, pH 7.2) and asymmetric (250/20 mM KCl, 10 mM MOPS/Tris, pH 7.2) conditions (Fig. 5). Under both conditions, the *I–V* relationship was highly non-linear, with the current amplitude being dramatically reduced at positive, compared to negative, voltages. This implies that the open probability of the HypF-N pore is voltage-dependent. Hence, under physiological conditions present in the IMM (with an inner membrane potential of around − 150 mV), the HypF-N pores would most likely be in the open state. It is possible to determine the cation or anion selectivity of a pore by measuring the *E*_*rev*_ (i.e. the electrical potential at which net current is zero) in asymmetric conditions. The observed *E*_*rev*_ in 250/20 mM KCl was + 15 mV, suggesting that HypF-N pores are selective for cations.

### Detailed analysis of conductance events and dwell-times

**Figure 6:**
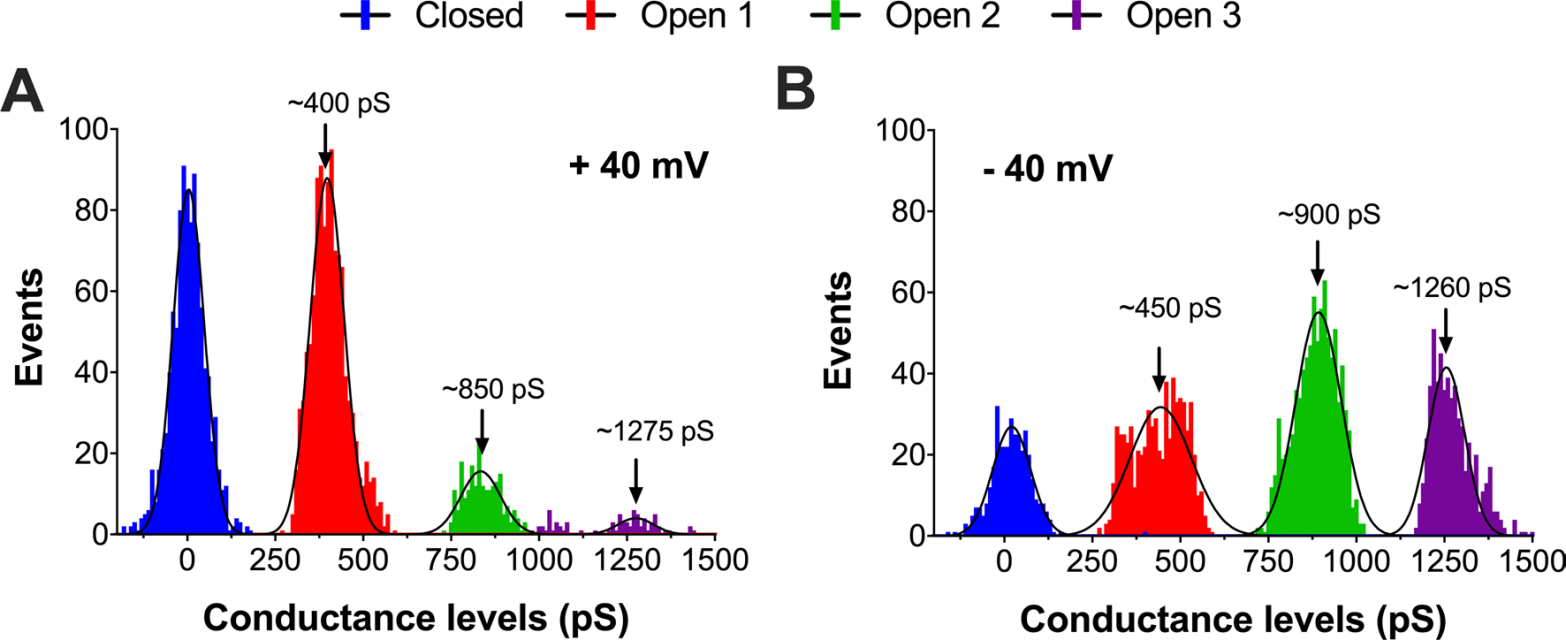
Histograms of conductance levels by HypF-N type A oligomers recorded in IM-type bilayers. (**A**) Histogram analysis of all the closed and multi-level open conductance states at + 40 mV (*n* = 3349) and − 40 mV (*n* = 3349) applied holding potential from a single-channel recording of a detected pore incorporated in IM-type planar BLM. Conductance histograms are fitted to a Gaussian distribution (solid black lines) with assigned peaks indicated by arrows. Peak conductance levels differ from each other by ~ 400–450 pS, indicating quantisation of conductivity with a smallest step of ~ 400–450 pS. Electrical recordings were carried out in 10 mM MOPS/Tris and 250/250 mM KCl, pH 7.2 at 22 °C.

At this stage, a quantitative analysis of the distribution of conductance steps and conductance levels recorded in the current traces obtained from type A HypF-N oligomers under symmetric conditions (250/250 mM KCl, 10 mM MOPS/Tris, pH 7.2) was undertaken. Evidence of well-defined conductance behaviour would be typical of the incorporation of a protein-based nanopore structure in the lipid bilayer^50,51^. Histograms of the measured conductance levels were constructed and the data fitted into a Gaussian distribution with three separate peaks (excluding the baseline) at both positive (+ 40 mV; Fig. 6A) and negative (− 40 mV; Fig. 6B) applied holding potential. At + 40 mV, peaks occurred at ~ 400 pS (Open 1), ~ 850 pS (Open 2) and ~ 1275 pS (Open 3). At − 40 mV, peaks occurred at almost identical conductance levels: ~ 450 pS (Open 1), ~ 900 (Open 2) and ~ 1260 pS (Open 3). Hence, type A HypF-N oligomers are indeed able to form high-conductance (hundreds of pS) membrane-spanning pores. Moreover, it was noted that the three peak conductance levels showed evidence of quantisation, with a first peak at ~ 400–450 pS (Open 1) and subsequent peaks at approximately double and triple that value. This quantisation phenomenon possibly infers fixed structural arrangements within the same pore unit (Open 1 ↔ Open ↔ 2 Open 3) or opening/closing events of up to three uniform (Open 1) pores. The availability of peak conductance levels for Open 1–3 allowed us to estimate the pore diameters, assuming a simplified cylindrical hole for a pore according to the model developed by Hille and adapted by Cruickshank^52^. Considering a membrane thickness of 7 nm for the IMM^36^, the calculated pore sizes for the three defined peak conductance states range between 1.2 and 2.2 nm (Table 1).

**Table 1:** Estimated conductances and pore diameters for type A HypF-N oligomers.

Level	Conductance (pS)	Pore diameter (nm)
1	~ 400	1.2–1.3
2	~ 850	1.8
3	~ 1275	2.2

This table shows the approximate diameter of a HypF-N pore calculated from the peak conductances at Levels 1, 2 and 3. It is important to note that Level 2 and Level 3 conductances may also represent the simultaneous opening of two or three Level 1 HypF-N pores, respectively.

The histograms also demonstrate that, while the greatest number of conductance events at + 40 mV occurred at Open 1 (Open 1: 80% of total events; Open 1: 10,649, Open 2: 2204, Open 3: 509), at − 40 mV almost the same share of events was represented by the Open 2 and Open 3 states together (Open 2 + Open 3: 75% of total events; Open 1: 6933, Open 2: 8909, Open 3: 5432). Further, significantly more (~ 1.6-fold higher) events occurred at negative than positive voltages (21,274 events at − 40 mV vs*.* 13,362 events at + 40 mV). The overall inference is a clear preference of HypF-N pores for membrane-pore forming activity in negatively-charged membranes.

**Figure 7:**
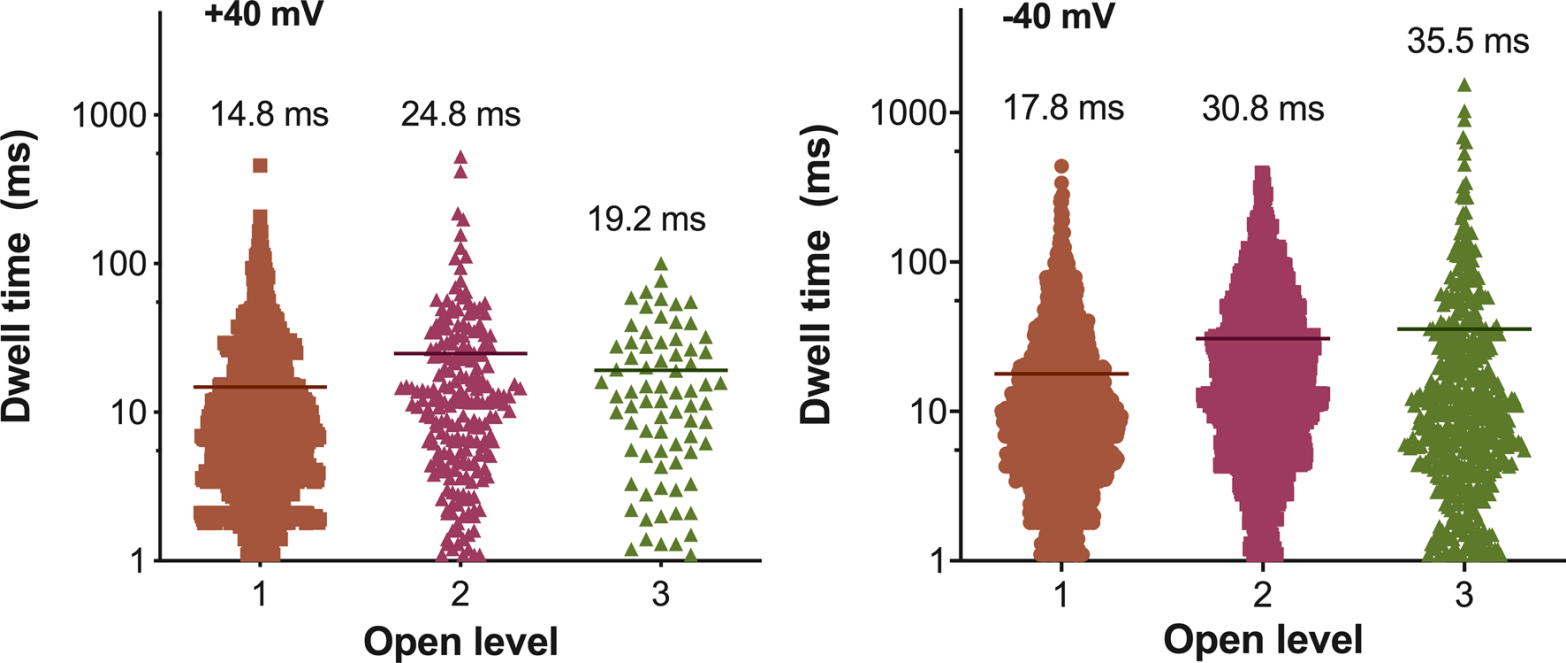
Dwell-times for HypF-N type A oligomers at defined conductance states Open 1 to 3. Scatter plot of dwell-times recorded at conductance states Open 1, 2 and 3, at + 40 mV (*left panel*) and − 40 mV (*right panel*). The mean dwell-time (in ms) and its s.e.m. is also shown for each Open level. At a negative membrane potential, the open probability of Open 2 and Open 3 states is markedly increased.

We also looked at the dwell-times of the three Open states, calculating the range and mean dwell-time of each (Fig. 7). There were no large differences between the mean dwell-times at positive or negative voltages, except for an appreciable increase in the dwell-time of the Open 3 state at − 40 mV (from 19.2 ms at + 40 mV, to 35.5 ms at − 40 mV). The overall range for the dwell-times is also highest for the Open 3 state at − 40 mV, with Open 3 activity persisting for even up to ~ 1.5 s (Fig. 7). Thus, the inference is that of longer periods of conductivity in negatively charged membranes, either by a larger Open 3 pore or when three Open 1 pores are concurrently active.

Taken together, our electrophysiological data led us to hypothesise a model in which type A HypF-N oligomers form high-conductance, cation-selective membrane-spanning pores in mitochondrial-like planar bilayers. Moreover, at negative membrane voltages, ionic current events tend to be more elevated and prolonged. Pores greater than 0.3 nm diameter as those observed here would be large enough to allow passage of water molecules, while most types of ions would be able to pass through a pore with a diameter of ~ 1.2 nm.

## Discussion

The current work focused on gaining insight into the disruptive effects of aggregates of the HypF-N protein on mitochondrial membranes, exploiting an especially experimentally-useful feature of the HypF-N model, namely that two confomers of morphologically identical HypF-N oligomers (type A and type B) can be easily generated^11–13,53–55^. We thus applied three different cell-free systems that manifest physiologically-relevant lipid composition, to study the effects of HypF-N on mitochondrial membranes: synthetic LUVs composed of lipid constituents that mimic those of mitochondrial (IM-type) and synaptic (C-type) membranes, freshly isolated mitochondria from a human neuroblastoma cell line, and an artificial planar bilayer constituted of IM-type lipids for single-channel electrophysiology. Together, these three in vitro systems were all concordant in demonstrating that toxicity to mitochondrial membranes is highly specific to type A HypF-N oligomers. As a matter of fact, to our knowledge, this study provides the first conclusive evidence supporting the formation of ion-conducting pores by pre-fibrillar aggregates of an amyloidogenic protein not associated with disease, in membranes mimicking the IMM or mitochondrial contact sites. Neither type B HypF-N oligomers nor HypF-N in its native monomeric state caused any appreciable leakage from lipid vesicles, any deleterious effects on mitochondrial function, or pore-like electrical activity in planar membranes. The latter observation is important, because despite their similar size and morphology, only type A oligomers have been found to display significant toxicity in cultured neuronal cells, cultured primary neurons, and whole animal models^13,14^. Presumably, the high surface hydrophobicity of type A oligomers^11,12,54^ encourages promiscuous interaction not only with cellular but also mitochondrial membranes, which leads to poration and permeabilisation of the latter.

It had been first reported by Relini et al*.* that pre-fibrillar aggregates of HypF-N insert into, and permeabilise, synthetic phospholipid bilayers^56^. We corroborated these findings by showing a greater vulnerability of mito-mimetic membranes to HypF-N-induced permeabilization. Of note, mito-mimetic lipid vesicles were not only significantly more perturbed by type A oligomers, but also by protofibrillar and even native (monomeric) HypF-N. In the latter case, it is likely that in situ amyloid aggregates of HypF-N were generated by adsorption and aggregate nucleation of HypF-N monomers at the lipid vesicle interface, culminating in membrane destabilisation^57^.

Generally speaking, mitochondrial membrane disruption by toxic HypF-N species could occur through either (i) a membrane thinning process as a result of lateral separation of lipid headgroups by the peptides, (ii) generation of protein-stabilised pores (poration), and/or (iii) a detergent-like mechanism with removal of lipid components from the bilayer^58,59^. The observed two-step kinetic profile for dye release in the LUV experiments is customarily attributed to the formation of pores (first step) and to bilayer disruption by a detergent-like mechanism (second step). Intriguingly, such two-step processes for membrane perturbation have been similarly proposed for Aβ- and IAPP-induced membrane disruption^42,60^. The single-channel electrophysiology recordings compare favourably with a process involving formation of HypF-N-induced pores in mito-mimetic membranes. Two distinct conductance behaviours could be distinguished, wherein type-I pores were characterised by fast transitions between low and high conductance states, while type-II pores were represented by step-like fluctuations with a reasonably well-defined open pore conductance. The flickering electrical activity of type-I pores, coupled with the fact that, when observed, they always preceded type-II pores, seems to indicate that the former may represent an intermediate, transient state which stabilises into a more robust ion-conducting structure. Electrophysiological characterisation of pores formed by Aβ_42_ oligomers in lipid bilayers reported a similiar observation of heterogeneous current behaviour, with combined rapid flickering and nanopore-like currents^49,61^. In our study, analysis of the current traces revealed peak conductance levels and steps (at − 40 mV: *G*_Open 1_ = 450 pS, *G*_Open 2_ = 900 pS and *G*_Open 3_ = 1250 pS) with longer periods of conductivity at the negative voltages. In particular, having distinct conductance *G* levels that are close multiples of a basic (quantum) event (i.e. *G*_Open 2_ = *G*_Open 1_ × 2 and *G*_Open 3_ = *G*_Open 1_ × 3) makes a convincing case for a HypF-N pore being formed by fixed protein subunits, either as a single pore changing between three conformational states or as up to three uniform pores fluctuating between ‘open’ and ‘closed’ states. The present study was based on other, similar, electrophysiology bilayer studies that looked at amyloid proteins related to neurodegenerative diseases, such as the Aβ peptide, α-synuclein and tau, with analogous observations^62^. For instance, Bode et al*.*^51^ reported that Aβ_42_ oligomers in membranes excised from HEK293 immortalised cells formed three distinct channel structures that transiently flickered between multiple conductance states (310–642 pS) and suggested that the channels were dynamic structures that could change in pore dimensions. Tosatto et al*.*^63^ detected three conductance states for α-synuclein in planar BLM (35 pS, 340 pS and 1020 pS), corresponding to barrel-stave pores formed by tetrameric clusters of α-synuclein molecules. Of even greater relevance to the present investigation, we recently reported on single-pore recordings by early aggregates of α-synuclein and tau in the same lipid environment as used in the current study (i.e. IM-type) exhibiting distinct ionic current events with open/close step transitions^64,65^. In this context, we speculate that a prominent pathogenic mechanism in neurodegenerative proteinopathies could proceed via the formation of ion-conducting pores in the membranes of mitochondria, impairing mitochondrial function and leading to neuronal demise.

The deleterious effects to mitochondrial integrity and function that could directly result from the formation of HypF-N nanopores in mitochondrial membranes were also evaluated on isolated mitochondria. We observed a combination of mitochondrial shrinking (loss of mitochondrial volume), partial loss in mitochondrial membrane potential, and a robust release of cytochrome *c* from mitochondria exposed to type A HypF-N protein aggregates. The electrophysiology data can further assist here in gaining mechanistic insight. Firstly, a higher activity and open probability at negative transmembrane potentials increases the likelihood of open high-conductance (100′s of pS) HypF-N pores in viable mitochondria, which typically have an inner membrane potential of around − 150 mV^66^. Secondly, the estimated dimensions of the main conductance states of HypF-N pores (1.3–2.2 nm for Open 1–Open 3) would be sufficiently large to permit the passage of most types of ions. In essence, a short-circuiting of the mitochondrial membrane potential would occur, and in agreement with this a lowering of the membrane potential of isolated mitochondria induced by HypF-N type A oligomers was observed. It has been calculated that opening of a single channel of the PorB porin of *Neisseria gonorrhoeae* with a main conductance state of ~ 400 pS, would lead to complete loss of the ΔΨ_m_ in about 0.8 ms^67^. A larger (~ 3 nm) pore would be required to allow the passage and efflux of cytochrome *c* from mitochondria. In this study, we showed that HypF-N type A oligomers allowed the gradual and size-dependent release of 40 kDa and 250 kDa FITC-dextran molecules from liposomes, suggesting the formation of large extended openings of defined size. Possibly, coalescing of the smaller HypF-N channel-like pores leads to larger pores in the membrane, as in the formation of supramolecular openings in mitochondrial outer membrane by the pro-apoptotic Bax and Bid peptides^68^. Lastly, another important consequence upon addition of type A HypF-N aggregates was the induction of a ~ 30% shrinking of the mitochondria in the sample. Although mitochondria typically swell before apoptosis, mitochondrial shrinking may cause an inhibition of respiration because flow of electrons from complex I and complex II into complex III is strongly inhibited by a decrease in matrix volume^43,69^. Shrinking can be ascribed to outward flow of water from the mitochondrial matrix, following the translocation of osmotically active solutes. For instance, efflux of K^+^ ions from the matrix, mediated by the K^+^ ionophore nigericin, has been shown to cause shrinkage of mitochondria^70^. In addition, a slightly positive reversal potential of the HypF-N pore under asymmetric conditions (+ 15 mV) was found, meaning that cations such as Na^+^, K^+^ or Ca^2+^ ions would in fact be allowed a greater degree of flux relative to anions such as Cl^−^ ions.

In summary, our mitochondria-based findings complement the results obtained with artificial membrane systems (LUVs and planar bilayer membranes) and lend further support to the general postulate that membrane-active aggregates of amyloid-forming proteins possess an inherently elevated toxicity to mitochondrial membranes. Perhaps a greater exposure of membrane acyl chains due to high negative curvature imparted by CL molecules facilitates the formation of strong hydrophobic interactions with exposed hydrophobic residues on the surface of the toxic oligomers, thereby accommodating insertion and promoting pore formation^71,72^. Through a detailed and rational analysis of 12 oligomeric variants of HypF-N and by comparing toxic/non-toxic oligomeric confomers of amyloid-β and α-synuclein, it was concluded that exposure of hydrophobic patches on the surface of a misfolded protein oligomer appears to be the structural factor most closely associated with oligomer toxicity^54,73–75^. Having said that, the possibility remains that increased membrane affinity of the type A HypF-N oligomers may also depend on specific conformations. For instance, membrane-affinity and toxicity of amyloid-β oligomers has been associated with acquisition of a specific tertiary fold during monomer to oligomer transition^76^. Thus, application of Raman spectroscopy and lipid-coated nanoparticles revealed the presence of β-turns flanked by antiparallel β-sheets in the membrane-bound amyloid-β oligomers^77^. Interestingly, such β-sheet–β-turn–β-sheet architecture allows for a β-barrel structure characteristic of transmembrane porins^78^. Moreover, free lipids may also mediate mechanisms of membrane poration by amyloidogenic peptides/proteins, as it was shown they can facilitate their insertion into membranes via ‘lipid-chaperoning’^79^.

In a general sense, our conclusions now support and extend this notion to oligomer interaction with mitochondrial membranes, thus representing a further conceptual advance on the cytotoxic role of small soluble oligomers in neurodegenerative diseases. It follows that therapeutic strategies which target the interaction between protein aggregates and mitochondrial membranes, or that reinforce the mitochondrial membrane against its oligomer-mediated disruption or poration, might represent a promising avenue for drug development for treatment of protein misfolding disorders^80,81^.

## Methods

### Preparation of HypF-N protein and aggregation

Expression and purification of recombinant HypF-N protein from an *E.coli* expression system were carried out as reported previously^11^. Aliquots of HypF-N were stored in LoBind tubes at − 20 °C in 20 mM phosphate buffer with 2 mM dithiothreitol (DTT) at pH 8.0. Upon thawing, aliquots were centrifuged for 10 min at 15,000 × *g*. Native HypF-N was aggregated into stable oligomeric (type A or type B) or protofibrillar species following established protocols^10,11^. In brief, oligomeric aggregates of HypF-N were prepared by incubating 48 μM protein for 4 h at 25 °C, either (a) in 50 mM sodium acetate buffer, 12% (v/v) tetrafluoroethanol (TFE) and 2 mM DTT, pH 5.5 (condition A), or (b) in 20 mM trifluoroacetic acid (TFA), 330 mM NaCl, pH 1.7 (condition B). Protofibrillar HypF-N was prepared by incubating 28 μM protein for 48 h at 25 °C in 50 mM sodium acetate buffer, 30% (v/v) TFE, pH 5.5 (condition P). For the mitochondria experiments, the oligomers were centrifuged at 16,100 × *g* for 10 min, dried under N_2_ gas and immediately resuspended in mitochondrial storage buffer.

### Preparation of large unilamellar lipid vesicles (LUVs)

Chloroform solutions of the phospholipids l-α-phosphatidylcholine (egg PC), l-α-phosphatidylethanolamine (egg PE), l-α-phosphatidylinositol (soy PI), l-α-phosphatidylserine (brain PS), heart cardiolipin (CL), and a mixture of 1,2-dioleoyl-sn-glycero-3-phosphoethanolamine (DOPE):1,2-dioleoyl-sn-glycero-3-phospho-l-serine (DOPS):1,2-dioleoyl-sn-glycero-3-phosphocholine (DOPC) (5:3:2, w/w/w) were purchased from Avanti Polar Lipids, Inc. (Alabaster, AL, USA). To prepare mitomimetic LUVs, required quantities of the lipids were mixed at defined proportions (% by weight of total phospholipids) on the basis of published data^37,82^. Thus, the following multicomponent lipid membrane (IM-type) resembles the phospholipid composition at the IMM and at mitochondrial contact sites: 45% PC, 25% PE, 10% PI, 5% PS and 15% CL. Another type of LUV (C-type) was prepared having a synthetic phospholipid blend which lacks the mitochondrial signature phospholipid cardiolipin and is representative of synaptic vesicle and neuronal membranes^83^: 50% DOPE, 30% DOPS, 20% DOPC. After aspirating the chloroform solvent under vacuum, the dry lipid film was suspended by vigorous vortexing in an aqueous liposome buffer (LB: 100 mM KCl, 10 mM MOPS/Tris, 1 mM EDTA, pH 7.0) containing either 50 μM OG or 2 mg/ml FITC-dextran. The glass tube containing the lipid suspension was covered in aluminium foil and left standing at room temperature for 60 min. Thereafter, 60 μl of 0.8 M Mega-9 detergent was added to the lipid suspension and vortexed repeatedly for a few seconds until a clear solution was seen. To eliminate any external free OG dye, the dialysis method was used^84^, while external free FITC-dextran was removed by 3–4 cycles of centrifugation. Liposome sizing was performed by dynamic light scattering (DLS) measurements using a Zetasizer Nano-ZS (Malvern Panalytical, Malvern, Worcestershire, UK). On average, liposomes were found to have a diameter of ~ 120 nm.

### Membrane leakage assays

To monitor the permeation of LUVs, 50 μM OG-loaded liposomes in LB containing 1 mM CaCl_2_ or 50 μM FITC-loaded liposomes were pipetted into solid black 96-well plates. In the case of OG-loaded liposomes, intravesicular OG was sequestered from external Ca^2+^ ions, but with addition of permeabilising agent or protein causing increased membrane leakage and a robust increase in fluorescence from binding of the BAPTA moiety of OG to Ca^2+^ ions. In the case of FITC-loaded LUVs, release of trapped FITC-dextran molecules upon membrane permeation results in decreased self-quenching with a consequent increase in fluorescence emission.

Measurements (in triplicate) were performed at 25 °C using a BioTek FLx800 microplate reader (exc. 485 nm, em. 528 nm). Permeabilisation of LUVs was calculated according to the equation:

$$ Permeabilisation \, \left( \% \right) \, = \, 100 \, \times \, \left( {F_{HypF - N} {-} \, F_{baseline} } \right)/\left( {F_{max} {-} \, F_{lipo} } \right) $$

where *F*_*HypF-N*_ denotes the maximum fluorescence intensity achieved during incubation of LUVs in LB with HypF-N protein aggregates; *F*_*baseline*_ represents the fluorescence of LUVs incubated in LB containing buffer alone for condition A, condition B or condition P; *F*_*lipo*_ represents the fluorescence of LUVs incubated in LB alone; and *F*_*max*_ was experimentally determined by disrupting the LUVs with the Ca^2+^ ionophore ionomycin (2 μM). Importantly, HypF-N protein aggregates alone carried no significant inherent fluorescence. Fluorescence intensity values are given in relative fluorescence units (RFUs).

### Mitochondrial isolation from SH-SY5Y cells

Mitochondria were isolated from SH-SY5Y human neuroblastoma cells (ECACC UK, Cat #94030304) using a MITOISO2 kit (Sigma-Aldrich, St Louis, Missouri, USA) to yield an enriched mitochondrial fraction with viable, intact mitochondria as previously described^84^. The mitochondrial pellet was resuspended in 1 × mitochondrial buffer (MB: 10 mM HEPES, 5 mM succinate, 250 mM sucrose, 1 mM ATP, 0.08 mM ADP, 2 mM K_2_HPO_4_, 1 mM DTT, pH 7.5) at 1–2 mg/ml. Mitochondria were kept at 4 °C during the entire isolation procedure, and only freshly prepared mitochondria were used for experiments.

### Absorbance measurements for mitochondrial volume

Freshly isolated mitochondria were brought to an initial A_540_ of ~ 0.35–0.40 in 1 × MB and the mitochondria allowed to acclimatise for ~ 15 min at 37 °C. Then, 250 μM CaCl_2_, 50 μM alamethicin or 10 μM HypF-N (in moles of monomeric or aggregated form) were added to the MB, and change in mitochondrial volume was monitored by taking absorbance readings using a spectrophotometer at 540 nm every 5 min for 60 min at 37 °C, from duplicate samples. The blank (buffer alone without mitochondria) was subtracted from all other values, and data expressed as means of absorbance readings ± s.e.m.

### Quantification of cytochrome *c* released from isolated mitochondria

Isolated mitochondrial suspensions (20 μg) were allowed to incubate at 30 °C for 1 h, alone or in the presence of HypF-N aggregate species. Maximal CCR was determined by adding the detergent Triton X-100 (1%, v/v). The total volume of the sample was kept at 100 μl. The samples were then centrifuged at 16,000 *g* for 10 min at 4 °C to collect the supernatant. The concentration of cyto *c* in supernatants was determined using the Quantikine Rat/Mouse Cytochrome *c* Immunoassay kit (MCTC0; R&D Systems) as per instructions. Absorbance at 450 and 540 nm was measured in a plate-reading spectrophotometer (BioTek ELx800, Winooski, VT, USA). The latter was subtracted from the former to correct for background absorbance. The resulting value was given as a percentage of 1% (*v/v*) TX-100).

### Measurement of the membrane potential of isolated mitochondria

Isolated mitochondria (equivalent to 5 μg protein) were stained with the JC-1 kit (Isolated Mitochondria Staining Kit, Sigma-Aldrich, St Louis, Missouri, USA) for measurement of the mitochondrial membrane potential (Δψ_m_) as previously described^65^. JC-1 fluorescence was monitored at 30 °C for 30 min, using 490 nm excitation and 590 nm emission in a BioTek FLx800 microplate reader (Winooski, VT, USA). Samples were tested in triplicate and the Δψ_m_ was obtained by subtracting the average carbonyl cyanide-p-trifluoromethoxyphenylhydrazone (FCCP) baseline, representing complete dissipation of the Δψ_m_, from the maximal JC-1 fluorescence measured. The obtained Δψ_m_ was given as a percentage of untreated mitochondria control (100%).

### Electrophysiological monitoring in a planar lipid bilayer system

The electrophysiological properties of HypF-N-induced pores were studied using an Ionovation Compact workstation (Ionovation GmbH, Osnabrück, Germany) connected to a HEKA EPC10 amplifier (HEKA Elektronik). Briefly, an artificial BLM was formed by ‘painting’ the lipid mixture across a 120 μm Teflon aperture separating *cis* and *trans* chambers filled with 250/250 mM KCl, 10 mM MOPS/Tris, pH 7.2. Bilayer formation was monitored optically using a dedicated CCD camera and by real-time capacitance measurements. Membranes that manifested instability, abnormal capacitance, or abnormal background conductance (> 15 pS) were discarded and replaced. In the event of an increase in bilayer conductance (threshold > 70 pS) and hence a pore event, automated pre-defined recording protocols were run by the Patchmaster software (HEKA, Germany) as previously described^64^. Ion selectivity was determined by changing to an asymmetrical salt concentration (250/20 mM KCl, *cis/trans*) to enable measurement of the reversal potential. All electrophysiology experiments were performed at room temperature (22 °C) and a trial typically lasted ~ 2 h. Digitised recordings were analysed using the Clampfit 10 software package (Molecular Devices). Further statistical analysis was done using GraphPad Prism 8 (GraphPad Software, Inc.).

### Estimation of pore diameter

The pore diameter was estimated from defined conductance levels assuming a simplified cylindrical nanopore, hence conductance *G* is directly proportional to the pore cross-sectional area. Under this assumption, the pore diameter (*2r*) can be calculated by the following equations^63^:

1 $$ r = r_{0} [{1 } + \, ({1 } + { 4}L/\pi r_{0} )^{{{1}/{2}}} ] $$

2 $$ r_{0} = G/{4}\sigma $$

where *r* is the pore radius, *G* the single pore conductance, *L* the pore length, and *σ* the bulk electrolyte solution conductivity (measured as 29.1 mS/cm at 22 °C for 250 mM KCl). We assumed *L* to be equal to the membrane thickness, i.e. 7 nm for mitochondrial membranes^36^.

### Statistical analysis

Statistical analyses and curve fitting were carried out using GraphPad Prism 8. Results are expressed as means ± s.e.m., with *n* as the number of independent experiments unless expressly indicated. Normality was assessed on all samples subjected to statistical analysis to ensure data met the assumptions of the tests used. Differences between means for more than two groups were determined by one-way or two-way ANOVA followed by Bonferroni’s post-hoc correction. For all analyses, a value of *p* < 0.05 was considered to be significant; exact *p* values are given whenever possible.

## Data Availability

All data are available from the corresponding author upon reasonable request.
